# Plasmacytoid Dendritic Cells and ICOS^+^ Regulatory T Cells Predict Poor Prognosis in Gastric Cancer: A Pilot Study

**DOI:** 10.7150/jca.34826

**Published:** 2019-10-22

**Authors:** Xiaosun Liu, Hang Yu, Chongxian Yan, Ying Mei, Caizhao Lin, Yanyun Hong, Xianke Lin, Qing Zhang, Jiren Yu

**Affiliations:** 1 Department of Gastrointestinal Surgery, The First Affiliated Hospital, Zhejiang University School of Medicine, Qingchun Road 79, Hangzhou, 310003, China; 2 Department of Colorectal surgery, The First Affiliated Hospital, Zhejiang University School of Medicine, Qingchun Road 79, Hangzhou, 310003, China

**Keywords:** Gastric cancer, Prognosis, Regulatory T cell, Plasmacytoid dendritic cell

## Abstract

**Background:** Regulatory T cells (Tregs) and plasmacytoid dendritic cells (pDCs) are the main immunosuppressive cells in tumor microenvironment of gastric cancer (GC). In this prospective study, the association of prognosis with Tregs subsets and pDCs were further analyzed.

**Methods:** pDCs, Tregs population and its expression of inducible costimulator (ICOS) were analyzed in peripheral blood from 41 GC patients by multicolor flow cytometry. These cell populations in carcinoma tissue, peritumor tissue and normal gastric mucosa from 87 GC patients were also detected by immunohistochemistry and double immunofluorescence.

**Results:** Both ICOS^+^Foxp3^+^Treg cells (P=0.0341 and P=0.0298, respectively) and pDC (*P*=0.0237 and *P*=0.0083, respectively) in peripheral blood and tumor tissue could predict poor clinical outcome in GC patients. However, the total Foxp3^+^Tregs in the GC tissue didn't correlated with the outcome (*P*=0.4299). No correlation of CD4^+^ T cell or CD8^+^ T cell frequency could be found with clinical outcome neither in peripheral blood nor in tumor tissue.

**Conclusions:** ICOS^+^Tregs and pDCs could predict poor prognosis of GC, targeting ICOS-L/ICOS costimulation axis may be a potential treatment for GC.

## Introduction

Approximately one million gastric cancer (GC) cases are estimated to occur every year, leading to the fifth most widespread tumor in the world and the third most common cause of cancer-related deaths worldwide currently 1. Overall survival for patients with GC remains poor. Tumor immune escape plays an important role in the recurrence and metastasis of gastric cancer. Thus, understanding immune mechanisms underlying therapeutic success and failure has important clinical relevance.

Plasmacytoid dendritic cells (pDCs) are one of the unique DC subsets and being extensively studied in innate immunity. pDCs express CD123, CD4, HLA-DR, blood-derived dendritic cell antigen-2 (BDCA-2), BDCA-4 and toll-like receptor (TLR) 7 and TLR9, but negative for lineage and CD11. pDCs play a major role in anti-viral responses, through their unique capacity to produce massive amounts of IFN-a in response to viral nucleic acids 2. However, their role in malignancy has not been well clarified. pDCs have been found in many tumor micro-environment, including gastric cancer, epithelial ovarian cancer, melanoma, head and neck cancer, breast cancer and lung cancer^3^-^8^. Their interaction with tumor cell and micro-environment appears to contribute to immunologic tolerance rather than anti-tumor effect. In fact, the number of pDCs were associated with disease progression and poor prognosis in several tumors, including ovarian cancer and breast cancer^3^,^6^.

Although not completely understood, pDC tolerance promotion by activating regulatory T cells (Tregs) had been proposed to explain the associated pDC contribution to immune tolerance in different cancers. Tregs are critical in the maintenance of immune tolerance and involved in suppressing deleterious immune responses to the host. Evidence suggested Tregs were immuno-suppressive lymphocytes that contributing to immune escape and suppressing anti-tumor immune response^9^,^10^. According to whether express inducible costimulator (ICOS), Tregs can be divided into ICOS^+^ Tregs and ICOS^-^ Tregs. The functions of these two subsets were different: ICOS^+^ Tregs secrete much larger amounts of interleukin 10 (IL-10), a critical negative regulator in tumor escape; while ICOS^-^ Tregs have a high capacity for TGF-β expression^11^. Indeed, the major Treg subset in tumor expressed ICOS, such as papillary thyroid cancer and GC, as we reported previously^9^,^10^. The number of ICOS^+^ Tregs were always associated with disease progress and early relapse^3^,^12^.

We previously have revealed that there were more pDCs and ICOS^+^ Tregs in GC patients both in circulating and tumor tissue when compared with health population and ICOS^+^ Tregs in stage III and IV patients' tumor and peritumor tissue were significantly increased than that of stage I and II patients'. However, the prognostic values of ICOS^+^ Tregs and pDCs had not been revealed. In fact, to the best of our knowledge, there were no studies correlating pDCs with clinical outcome in GC. Here, we extend and validate these results by using two different and complementary approaches (flow cytometry and immunohistochemistry) on two independent cohorts of GC patients.

## Materials and Methods

### Study subjects

The inclusion criterial were: 1) diagnosed with GC by gastroscopic biopsy; 2) received effective resection. The exclusion criterial were: 1) concurrent autoimmune disease, HIV, or syphilis; 2) concurrent chronic infection; 3) patients who received radiotherapy or chemotherapy before surgery; 4) history of other malignancies.

### Blood samples

As reported previously, 51 patients who were diagnosed with GC by gastroscopic biopsy were enrolled in this study. Four patients didn't receive operation after chemotherapy; six patients gave up on any treatment and thus ten patients were eliminated. So, 41 cases were enrolled.

### Tissue samples

91 patients with gastric cancer were enrolled for tissue samples, all of whom underwent surgery between 2007 and 2011. Four patients were lost to follow-up in the at least five years' follow up and 87 cases were enrolled in this study.

This study was approved by the ethical committee of the First Affiliated Hospital, Zhejiang University School of Medicine, and informed consent was provided by each people recruited to the study.

### Flow cytometry analysis

Multicolor flow cytometry was performed on fresh Ficoll-prepared (TBDsciences, China) PBMCs as described previously 9.

### Immunohistochemistry (IHC) and Immunofluorescence

As our previously published study described, BDCA-2 analysis was performed on frozen sections as the available anti-BDCA-2 antibody was not suitable for paraffin-embedded archive material during the experiment period 9. For Foxp3 and ICOS, double-fluorescence immunostaining was performed as described previously 9.

### Quantifying immunostaining parameters

Data were obtained by manually counting positively stained cells in 10 representative fields of normal mucosa, peritumor mucosa and cancer tissue under 400X high-power magnification. Double-fluorescence immunostaining was also determined by manual counting of positive cells in 10 high-power fields (HPF). Densities were determined by computing the mean number of positively stained cells per HPF. The median values were used as cut off.

### Follow-up

Complete follow-up was available for all patients. The last follow-up was in July 2018. Median follow-up was 76.5 months. For all the 128 patients, 44 patients were dead, 84 were still survival. 3- and 5-year overall survive (OS) was estimated to be 77.0% and 66.3 %, respectively.

### Statistical analysis

The end point was OS, which was calculated from the date of surgery to the date of death of any cause or last follow-up time, censoring patients who were alive. The Kaplan-Meier model was used for OS analysis. P value < 0.05 was taken to indicate statistical significance. Analysis was performed using GraphPad Prism 5.01 (GraphPad Software, San Diego, CA, USA) software.

## Results

### Patients

The clinical characteristics of the patients who provided blood (n=41, male=29, female=12) or tissue samples (n=87, male=62, female=25) were shown in **Table 1**. Of note, 30 tissue samples were randomly selected from a total of 87 tissue samples for immunofluorescence analysis. As for the 30 cases, 17 were male and 13 female; the mean age was 62±10 years; the TNM stages were as follows: stage I, 4; stage II, 7; stage III, 16; stage IV, 3.

### No Correlation of CD4^+^ T cell or CD8^+^ T cell frequency with clinical outcome both in blood and tissue

As our previous reported which was published in Cancer Science 2014 February, CD4^+^ T cell and CD8^+^ T cell were identified by IHC in tissue samples. Here in, the association of their frequency and prognosis were analyzed. The median values were used as cut off. Neither CD4^+^ T cell nor CD8^+^ T cell could predict the prognosis (P=0.9032 and P=0.0701, respectively), although there was a trend for higher CD8^+^ T cell numbers in carcinoma tissue correlated with a positive prognosis.

CD4^+^ T cell and CD8^+^ T cell in peripheral blood were recognized as CD3^+^CD4^+^ T cell and CD3^+^CD8^+^ T cell by flow cytometry. CD4^+^ T cell and CD8^+^ T cell in peripheral blood didn't show any relation with the prognosis (P=0.346 and P=0.9657, respectively).

### ICOS^+^Foxp3^+^Treg cells and pDCs predict poor clinical outcome in GC patients

**Figure 1:**
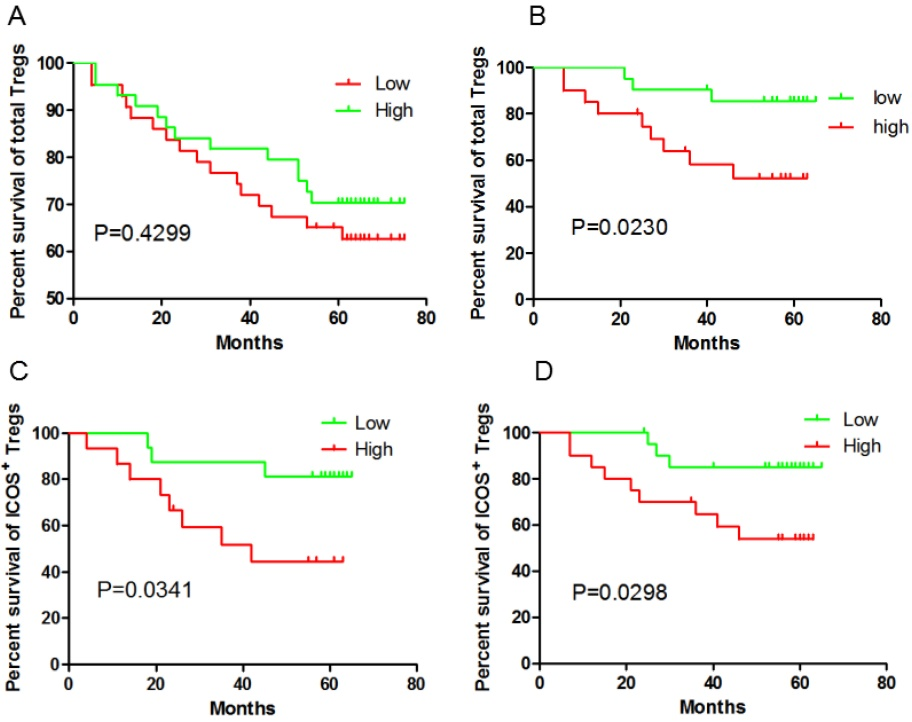
ICOS^+^ Tregs predict poor survival in gastric cancer patients. A and B, higher total Foxp3^+^ Tregs frequency in peripheral blood is correlated with the outcome, but not in GC tissue; C and D, both ICOS^+^ Tregs in peripheral blood and GC tissue predict poor survival.

Given our previous study had shown Treg cell-driven immunosuppression were linked to disease progression in GC, we questioned the impact of the presence of Tregs in tumors and blood on patients' outcome. The unexpected outcome was the total Foxp3^+^Tregs in the GC tissue didn't correlated with the outcome (P=0.4299, **Fig 1A**), while higher Foxp3^+^ Tregs frequency in peripheral blood predict a poor prognosis (P=0.023, **Fig 1B**). Foxp3^+^Tregs could be divided into ICOS^-^Foxp3^+^ and ICOS^+^Foxp3^+^. It was reported that ICOS^+^Foxp3^+^ Tregs were different from ICOS^-^Foxp3^+^ Tregs and considered as the main immunosuppressive cells. So in the next step, ICOS^+^Foxp3^+^Treg cells were analyzed. Both in the GC tissue ( 30 cases randomly selected from the total 87 tissue samples) and in peripheral blood, ICOS^+^Foxp3^+^ Tregs predict a poor clinical outcome (P=0.0341 and P=0.0298, respectively; **Fig 1C and 1D**). However, ICOS^-^Foxp3^+^ Tregs in GC tissue did not correlate with OS (P=0.137 ). Thus, our data indicate, at least in GC tissue, that ICOS^+^Treg cells but not ICOS^-^ Treg cells predict a poor clinical outcome.

**Figure 2:**
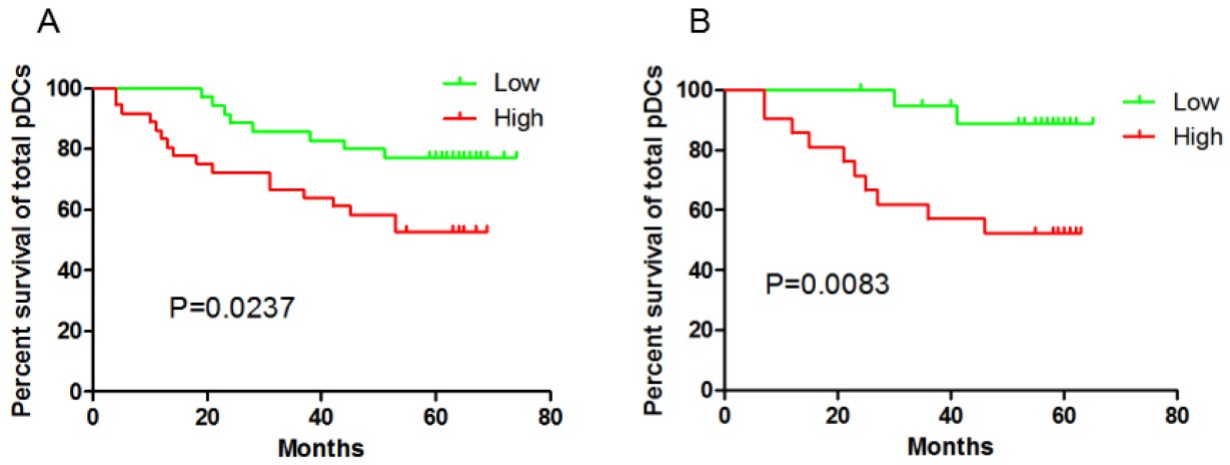
Both patients with higher pDCs numbers in GC tissue (A) and higher pDCs percentage in peripheral blood (B) suffered a shorter OS.

Immunohistochemical staining of tumor tissue have observed the significant correlation between pDCs and Treg cells infiltration in tissues^8^-^9^ . In this prospective study, higher BDCA-2^+^pDCs numbers in GC tissue and higher percentage of Lin^-^HLA-DR^+^CD11c^-^CD123^high^ pDCs /Lin^-^ cells in blood suffered a shorter OS (P=0.0237 and P=0.0083, respectively, **Fig 2**). In contrast, high percentage of mDC /Lin^-^ cells in peripheral blood had no impact on patients' outcome (date not shown).

## Discussions

In this prospective study on 41 GC patients providing blood samples and 87 providing tissue samples, we showed that ICOS^+^Foxp3^+^Treg cells and pDCs could predict poor clinical outcome. This work represented the prognostic value of ICOS^+^Foxp3^+^Tregs and pDCs both in tissues and peripheral blood in GC patients.

Most cancers, including GC, are highly infiltrated by immune cells. We previously have shown that the numbers of CD3^+^ T cells were similar in normal gastric mucosa, peritumor tissue, and GC tissue, CD4^+^ T cells were most abundant in GC tissue, then in peritumor tissue, gastric mucosa was the least. As for CD8^+^ T cells, the distribution was just the opposite to CD4^+^ T cells. High densities of CD3^+^ T cells, CD4^+^ T cells, CD8^+^ T cells in the tumor tissue have been were associated with better survival in GC, however, not independent prognostic factor 13. Our date showed no correlation of intratumoral CD4^+^ T cell or CD8^+^ T cell frequency with clinical outcome both in blood and tissue, which was in keeping with previous report 14.

Although the interaction of pDCs with tumor cells and their tumor immune micro-environment is complex, more and more evidence appears to show immunologic tolerance role rather than anti-tumour effect. In ovarian cancer, Sana Intidhar Labidi-Galy et al. found the presence of TApDC was associated with early relapse and the accumulation of pDC in tumors was an independent prognostic factor associated with short progression-free survival 15. Julien Faget et al. then highlighted an relationship between ICOS^+^ cells, Tregs and pDCs in breast tumors, and show that ICOS/ICOS-L interaction is a central event in immunosuppression of tumor-associated memory CD4^+^ T cells^12^. Curdin Conrad et al.'s found Foxp3^+^ Treg cells express ICOS and their expansion and the suppressive function were strictly dependent on ICOS-L costimulation provided by pDCs, ICOS^+^ Treg cell subset being a stronger predictor than total Foxp3^+^ Treg cells^3^.

We previously reported the increased numbers of total Tregs, ICOS^+^ Tregs and pDCs in peripheral blood of GC patients and Tregs, ICOS^+^ Tregs are accumulated in tumor tissue. Interestingly, pDCs are assembled in the peritumor tissue rather than in tumor tissue^9^. Herein, date indicated higher pDCs numbers in GC tissue and higher pDCs percentages in blood were associated with worse OS. To the date, no studies have focus on the pDC's impact on GC patients' outcome before. The only clue was a positive correlation of pDCs with ICOS^+^ Tregs in peritumor 8-9. Hirotsugu Nagase et al. speculated that the expression of ICOS in Foxp3^+^ Tregs was related to the expression of ICOS-L and TLR9 in pDCs as well as Helicobacter pylori infection^8^. The presence of mDC in blood had no impact on patients' outcome highlighting the specific pejorative role of pDC in the context of tumor microenvironment.

In our study, we revealed higher Foxp3^+^ Tregs in peripheral blood predict a poor prognosis. In gastric cancer, evidence are found that higher Foxp3^+^ Tregs in blood and GC tissue are associated with the shorter OS 13-14,16. However, as other paper reported, Foxp3^+^ Tregs in tissue didn't show predictive value 8. The limited cases in this paper may be one of the reasons. The total Foxp3^+^ Treg in tissue itself is not the best prognostic parameters, neither in ZB Shen's research nor in K Liu's, Foxp3^+^ Treg is not the independent predictor^13^-^14^. Unlike total Foxp3^+^ Treg, the predictive value of ICOS^+^Foxp3^+^ Tregs both in tissue and peripheral blood were more prominent. This was consisting with the results of anther research in GC. Given the limited samples for double-fluorescence immunostaining in tissue (30 cases) and blood (41 cases), our results indicate that ICOS^+^ Treg cells are a better predictor of disease progression than total Foxp3^+^ Treg cells.

In conclusion, ICOS^+^Foxp3^+^Treg cells and pDCs both in GC tissue and peripheral blood could predict a poor clinical outcome in GC. As a prognostic parameters, ICOS^+^ Treg cells maybe superior than total Foxp3^+^ Treg cells. Because of the unique relationship between pDCs and ICOS^+^ cells, targeting ICOS-L⁄ICOS costimulation maybe a potential treatment for GC.

## Figures and Tables

**Table 1:** Clinical characteristics of patients with gastric cancer

Factor		Blood sample	Tissue sample
		(n = 41)	(n = 87)
**Gender**	Male	29 (70.5%)	62 (71.3%)
	Female	12 (29.5%)	25 (28.7%)
**Age, years**	Male	59±11^*^	62±13
	Female	54±17	63±12
**Primary tumor** (T stage^**^)	T1	15 (36.6%)	25 (28.7%)
	T2	5 (12.2%)	28 (32.2%)
	T3	19 (46.4%)	21 (24.2%)
	T4	2 (4.8%)	13 (14.9%)
**Lymph node metastasis**	Negative	21(51.2%)	37 (42.5%)
	Positive	19(48.8%)	50 (57.5%)
**Distant metastasis**	Negative	40 (97.5%)	76 (87.4%)
	Positive	1 (2.5%)	11(12.6%)
TNM stage^**^	I	19 (46.3%)	27 (31.0%)
	II	3 (7.3%)	16 (18.4%)
	III	14 (34.1%)	31 (35.6%)
	IV	3 (7.3%)	13 (15.0%)

^*^ Age values are expressed as means±SD; ^**^ AJCC 6th edition

## Abbreviations

Tregs: regulatory t cells
pDCs: plasmacytoid dendritic cells
GC: gastric cancer
BDCA-2: blood-derived dendritic cell antigen-2
TLR: toll-like receptor
ICOS: inducible costimulator
IL: interleukin
IHC: immunohistochemistry
DAB: diaminobenzidine
OS: overall survival
